# A new *Rhodococcus aetherivorans* strain isolated from lubricant-contaminated soil as a prospective phenol-biodegrading agent

**DOI:** 10.1007/s00253-020-10385-6

**Published:** 2020-02-11

**Authors:** Taisiya Nogina, Marina Fomina, Tatiana Dumanskaya, Liubov Zelena, Lyudmila Khomenko, Sergey Mikhalovsky, Valentin Podgorskyi, Geoffrey Michael Gadd

**Affiliations:** 1 Zabolotny Institute of Microbiology and Virology of National Academy of Sciences of Ukraine, Zabolotny str., 154, Kyiv, 03143 Ukraine; 2 ANAMAD Ltd, Sussex Innovation Centre, Science Park Square, Falmer, Brighton, BN1 9SB UK; 3 Chuiko Institute of Surface Chemistry, 17, General Naumov Street, Kyiv, 03164 Ukraine; 4 Geomicrobiology Group, School of Life Sciences, University of Dundee, Dundee, Scotland DD1 5EH UK; 5 State Key Laboratory of Heavy Oil Processing, Beijing Key Laboratory of Oil and Gas Pollution Control, College of Chemical Engineering and Environment, China University of Petroleum, Beijing, 102249 China

**Keywords:** Phenol biodegradation, Actinobacteria, *Rhodococcus aetherivorans*, Cell immobilization, Xenobiotics, Bioremediation

## Abstract

Microbe-based decontamination of phenol-polluted environments has significant advantages over physical and chemical approaches by being relatively cheaper and ensuring complete phenol degradation. There is a need to search for commercially prospective bacterial strains that are resistant to phenol and other co-pollutants, e.g. oil hydrocarbons, in contaminated environments, and able to carry out efficient phenol biodegradation at a variable range of concentrations. This research characterizes the phenol-biodegrading ability of a new actinobacteria strain isolated from a lubricant-contaminated soil environment. Phenotypic and phylogenetic analyses showed that the novel strain UCM Ac-603 belonged to the species *Rhodococcus aetherivorans*, and phenol degrading ability was quantitatively characterized for the first time. *R*. *aetherivorans* UCM Ac-603 tolerated and assimilated phenol (100% of supplied concentration) and various hydrocarbons (56.2–94.4%) as sole carbon sources. Additional nutrient supplementation was not required for degradation and this organism could grow at a phenol concentration of 500 mg L^−1^ without inhibition. Complete phenol assimilation occurred after 4 days at an initial concentration of 1750 mg L^−1^ for freely-suspended cells and at 2000 mg L^−1^ for vermiculite-immobilized cells: 99.9% assimilation of phenol was possible from a total concentration of 3000 mg L^−1^ supplied at daily fractional phenol additions of 750 mg L^−1^ over 4 days. In terms of phenol degradation rates, *R*. *aetherivorans* UCM Ac-602 showed efficient phenol degradation over a wide range of initial concentrations with the rates (e.g. 35.7 mg L^−1^ h^−1^ at 500 mg L^−1^ phenol, and 18.2 mg L^−1^ h^−1^ at 1750 mg L^−1^ phenol) significantly exceeding (1.2–5 times) reported data for almost all other phenol-assimilating bacteria. Such efficient phenol degradation ability compared to currently known strains and other beneficial characteristics of *R*. *aetherivorans* UCM Ac-602 suggest it is a promising candidate for bioremediation of phenol-contaminated environments.

## Introduction

Phenols are the main pollutants of effluents and wastes of petrochemical, wood-chemical, pharmaceutical and plastics industries. Due to their high toxicity, even at low concentrations, ubiquity and resistance to degradation, they are considered to be particularly hazardous environmental pollutants (Gami et al. 2014; Paisio et al. 2014). A phenol concentration of 1 mg L^−1^ can affect aquatic life and be harmful to human health. The World Health Organization established a maximum permitted concentration of 1 μg L^−1^ for phenolic compounds in drinking water (Paisio et al. 2014). However, the purification of phenol-containing industrial wastewaters and contaminated environments is currently an important problem (Michalowicz and Duda 2007; Gami et al. 2014; Paisio et al. 2014). Physico-chemical methods such as reverse osmosis, ozonation, electrolytic oxidation and photocatalysis can be effective for phenol degradation in industrial wastewater (Lin and Chuang 1994; Shaban et al. 2013) but they are complex, expensive and frequently produce other toxic end-products, e.g. the conversion of phenol to chlorophenol when using chlorination (Marrot et al. 2006). Biological methods of phenol decontamination based on the use of phenol-biodegrading microorganisms may have advantages over physical and chemical treatments as they require relatively small investment and low power consumption as well as being capable of complete phenol degradation to carbon dioxide and water (Gami et al. 2014; Paisio et al. 2014). One of the flaws of conventional physico-chemical methods is that they are not able to reduce the phenol concentration to environmentally safe values. For example, physico-chemical treatment of contaminated industrial sewage at petrochemical enterprises by adsorption, evaporation and extraction reduces the phenol content from 2000 to 6000 mg L^−1^ to only 80–400 mg·L^−1^ which still requires additional post-treatment using biochemical methods to reduce the phenol content to 2–5 mgL^−1^ (Smirnova et al. 2017).

Phenol-utilizing microorganisms are capable of degrading phenol to non-toxic intermediates of the tricarboxylic acid (Krebs) cycle through *ortho-* or *meta-*cleavage pathways (Park et al. 2009; Paisio et al. 2014; Kim and Gadd 2019). To improve the efficacy of phenol degradation by microorganisms, many strategies have been proposed including an increase in size of the bacterial inoculum, co-metabolism by introduction of an additional conventional substrate (e.g. glucose or yeast extract), the use of bacterial consortia and immobilized cells as well as sequential fractional additions of substrate (Shumkova et al. 2009; Soudi and Kolahchi. 2011; Paisio et al. 2014; Al-Defiery and Gopal 2015). Bacterial species of the *Acinetobacter*, *Arthrobacter*, *Bacillus*, *Pseudomonas* and *Rhodococcus* genera are often identified as capable of phenol biodegradation (Paisio et al. 2014). However, only a few of them are able to metabolize the high phenol concentrations that can be found in anthropogenic wastes. According to several authors, one of the most promising groups of microorganisms suitable for bioremediation of phenol-polluted environments are actinobacteria of the genus *Rhodococcus* (Shumkova et al. 2009; Solyanikova et al. 2010; Suhaila et al. 2010, 2013; Soudi and Kolahchi 2011; Mishra and Lal 2014; Al-Defiery and Gopal 2015; Moghadam et al. 2016). The ubiquity of *Rhodococcus* spp. in nature, their broad catabolic versatility and physiological and ecological adaptability to extreme environmental conditions make them suitable candidates for biodegradation of persistent xenobiotics and bioremediation (Martínková et al. 2009; Kuyukina and Ivshina 2010; Solyanikova et al. 2010; Solyanikova and Golovleva 2015).

The majority of phenol-utilizing microorganisms are reported to grow at a maximum concentration of 600 mg L^−1^ (Paisio et al. 2014; Smirnova et al. 2017). Concentration values for phenol in anthropogenic sources show a wide range: phenol concentrations from petroleum refinery effluents vary between 50 and 2000 mg L^−1^, from distillation units and olive mill wastewater between 1200 and 4300 mg L^−1^ and from highly concentrated wastewater from coke-chemical and petrochemical enterprises between 5000 and 10,000 mg L^−1^ (Paisio et al. 2014; Smirnova et al. 2017). Phenol-containing wastewaters and contaminated soils are also usually accompanied by other industrial pollutants including oil hydrocarbons. Considering that the efficiency of microbial phenol degradation is strain specific, it follows that there is a need to search for new strains resistant to highly polluted environments and which are able to efficiently perform phenol biodegradation while withstanding variability in concentration and the presence of oil hydrocarbon co-contaminants. The aim of this work was therefore to study the phenol-biodegrading ability of an actinobacteria strain newly isolated from lubricant-contaminated soil.

## Materials and methods

### Chemicals

All chemicals used in this study were of analytical grade (Merck, Kenilworth, NJ, USA). Phenol was of chromatography grade (purity 99.5%, Merck). Commercial diesel fuel ‘Euro 5’ was obtained from PJSC Ukrtatnafta (Kremenchug, Poltava region, Ukraine), transformer oil ‘NITRO 11 GX’ from Nynas Naphthenics (Stockholm, Sweden) and expanded vermiculite (fraction size 4 mm) from the Ukrainian Vermiculite Group LLC (Vasil’kov, Kiev region, Ukraine). The chemical composition of vermiculite (%, expressed as oxides) was SiO_2_ (33–36); Fe_2_O_3_ (5–17); FeO (0.2–0.27); Al_2_O_3_ (6–18); MgO (14–25); CaO (1.2–2); K_2_O (3–5); Mn (0.05–0.07); Na_2_O (0.5); TiO_2_ (0.4–0.47); pH (H_2_O) 6.8–7.0 (http://uvg.org.ua/eng/descriptions/).

### Microorganisms

The bacterial strain was isolated from lubricant-contaminated soil at the locomotive depot of Odessa railway station (Odessa, Ukraine). A pure culture was obtained by the serial dilution (10^−1^) method followed by the spread plate technique using agar medium 53 (*Corynebacterium* agar) containing (L^−1^ distilled water): 10.0 g casein peptone, tryptic digest, 5.0 g yeast extract, 5.0 g glucose, 5.0 g NaCl, 15 g agar (DSMZ medium 53, www.dsmz.de). In addition, 200 mg L^−1^ phenol was added to the medium. The plates were incubated at 28 °C for 5 days. Morphologically different colonies were transferred onto agar medium 53 slants and the growth cycle of pure cultures examined using light microscopy after 16, 18, 24, 48 and 72 h of growth. The strain, UCM Ac-602, that showed a rod-coccus growth cycle was used for further studies. This strain was stored on agar medium 53 containing 200 mg L^−1^ phenol at 5 °C in the Ukrainian Collection of Microorganisms (UCM) of the Zabolotny Institute of Microbiology and Virology, National Academy of Sciences of Ukraine, Kyiv, Ukraine. Type strains of *Rhodococcus rhodochrous* UCM Ac-744 (= DSMZ 43241) and *Rhodococcus ruber* UCM Ac-745 (= DSMZ 43338) were obtained from the UCM to use as reference *Rhodococcus* species.

### Media and culture conditions

For chemotaxonomic studies, the organism was grown for 48 h in 750 mL Erlenmeyer flasks containing 100 mL of liquid agar-free medium 53 on a rotary shaker (220 rpm) at 28 °C. The biomass was harvested by centrifugation for 15 min at 5000 rpm, washed twice in distilled water and freeze-dried. For phylogenetic analysis, the cells were grown on agar medium 53, washed in NaCl/EDTA buffer (0.1 M EDTA, 0.1 M NaCl, pH 8.0) and stored until use at − 20 °C. Estimation of the degradation efficiency for oil hydrocarbons was performed using cells grown for 72 h in liquid mineral medium N1 containing (L^−1^ distilled water): 3.0 g KNO_3_, 0.28 g KH_2_PO_4_, 1.2 g Na_2_HPO_4_.12H_2_O, 2.0 g NaCI, 0.2 g MgSO_4_.7H_2_O, 17.9 mg CaCl_2_ · 6H_2_O, 19.9 mg FeSO_4_.7H_2_O, 1.0 g yeast extract, pH 6.8–7.0. Kerosene, n-hexadecane, diesel or transformer oil were added into the medium to 0.5% (*v*/*v*). A suspension of cells pre-grown on agar medium 53 for 48 h was used as an inoculum.

The experiments on growth and degradation of phenol by the bacterial strain were carried out in liquid mineral medium N2 containing (L^−1^ distilled water): 0.75 g NH_4_NO_3_, 0.73 g Na_2_HPO_4_, 0.35 g KH_2_PO_4_, 0.25 g NaHCO_3_, 0.1 g MgSO_4_.7H_2_O, 0.002 g MnSO_4_, 0.02 g FeSO_4_.7H_2_O; pH 7.0–7.2 (Shumkova et al. 2009). Phenol was filter sterilized using 0.2 μm regenerated cellulose membrane filters (Sartorius Stedim, Goettingen, Germany) and added, from 200 to 2000 mg L^−1^, to the autoclaved medium after cooling to room temperature. Bacterial growth was measured by optical density at 540 nm (OD_540_) using a photocolorimeter KFK-2MP (Zagorsk Optical and Mechanical Plant, Zagorsk, Russia). Phenol degradation by freely-suspended bacterial cells at initial substrate concentrations of 200, 300, 500 and 750 mg L^−1^ was studied by growing bacteria for 24 h. Samples were taken at regular intervals (2 h) and analysed for growth and phenol degradation. At the initial phenol concentrations of 1000 and 1500 mg L^−1^, the strain was cultivated for 72 h: at an initial phenol concentration of 1750–2000 mg L^−1^, it was grown for 144 h. Samples were taken every 8 h at phenol concentrations of 1000 and 1500 mg L^−1^ and every 24 h at concentrations of 1750 and 2000 mg L^−1^. Investigation of phenol degradation by immobilized cells was carried out in flasks containing 100 mL medium, 2.5 g vermiculite with immobilized cells and 2000 mg L^−1^ phenol. To test the ability of vermiculite to sorb phenol, an abiotic control without microorganisms in a medium with 2000 mg L^−1^ phenol and 2.5 g vermiculite was used. Culture conditions in this experiment were the same as for freely-suspended bacterial cells at 2000 mg L^−1^ phenol. In all experiments, a 48-h inoculum was used of bacteria grown on phenol-containing medium N2 with fractional phenol addition: initial cultivation was at a phenol concentration of 500 mg L^−1^ for 24 h which was then followed by the next phenol (500 mg L^−1^) addition and continued cultivation for 24 h. For determination of freely-suspended bacterial cell viability at an initial phenol concentration of 2000 mg L^−1^, samples were serially diluted and plated on agar medium 53. To study the effect of fractional phenol addition on degradation, an initial phenol concentration of 750 mg L^−1^ was used for 96 h in medium N2 with subsequent phenol additions to the culture every 24 h.

### Phenotypic properties

Cell morphology was examined using a Carl Zeiss Primo Star light microscope (Zeiss, Jena, Germany). Gram staining, motility, oxidase and catalase activity, gelatin and casein hydrolysis and nitrate reduction were tested using standard procedures (Smibert and Krieg 1994). For detection of the aromatic ring cleavage mechanism, the strain was grown for 24 h on medium N2 at an initial phenol concentration of 500 mg L^−1^. Cells were harvested from a 30 mL broth culture by centrifugation (5000 rpm, 15 min, 4 °C). The presence of the *β*-ketoadipate pathway (indicating *ortho*-cleavage) was suggested qualitatively by the Rothera reaction according to the method of Ottow and Zolg (1974). Other physiological and biochemical properties were determined as described by Goodfellow et al. (1990). Established methods were also used for detection of isomers of 2,6-diaminopimelic acid, analysis of whole-cell sugars (Staneck and Roberts 1974) and mycolic acids (Minnikin et al. 1980) using type strains of *R*. *rhodochrous* UCM Ac-744 and *R*. *ruber* UCM Ac-745 as controls. Fatty acid methyl esters in whole cells were prepared by cell hydrolysis in a 5% solution of acetyl chloride in methanol for 4 h at 100 °C followed by extraction with ether:hexane (1:1). Identification of methyl esters was performed using a gas chromatography-mass spectrometry system (Agilent 6800N/5973inert, Agilent Technologies, Santa Clara, CA, USA) and identified based on their retention time by comparison with standards. Fatty acid content was determined using Agilent Chem Station software and was expressed as a percentage (%) of total peak area.

### Phylogenetic analysis

Bacterial DNA was isolated from a 24-h culture using the DNA isolation kit ‘DNA-sorb B’ (AmpliSens, Moscow, Russia) according to the manufacturer’s instructions. 16S rRNA gene amplification was performed with primers 27f and 1492r as described by Lane (1991). PCR-product sequencing was carried out using ABI 310 (Applied Biosystems) with the ‘BigDye Terminator v3.1 Cycle Sequencing Kit’ (ThermoFisher Scientific, Waltham, MA, USA) and the 1492r primer. The resulted nucleotide fragment of the 16S rRNA gene sequence was compared with homologous sequences available in the GenBank database using the NCBI Blast program (http://www.ncbi.nlm.nih.gov/blast). Phylogenetic analysis was carried out using MEGA5 program (Tamura et al. 2011), a dendrogram was constructed with the neighbour-joining method and Kimura’s two-parameter model (Kimura 1980; Saitou and Nei 1987). Then, 1000-replica bootstrap was used for providing tree topology confidence. The 16S rRNA gene sequences of *Rhodococcus* species were obtained from the GenBank database and the web-resource www.straininfo.net.

### Determination of hydrocarbon and phenol degradation efficiency

The total amount of petroleum hydrocarbons was determined by IR spectrometry using a laboratory analyser of petroleum products in water AN-1 (Neftehimavtomatika-SPb, St Petersburg, Russia) according to the manufacturer’s instructions. The phenol concentration was determined by a direct photometric method using 4-aminoantipyrine according to the American Public Health Association (APHA) (2005). The hydrocarbon (h) or phenol (p) degradation efficiency (DE_h_ or DE_p_) was evaluated as a percentage (%) and calculated according to the formula: DE_h_ or DE_p_ (%) = 100% – [*C*_2_ × 100%/*С*_1_], where *C*_1_ is the primary hydrocarbon or phenol substrate concentration in a sample, mg L^−1^; *C*_2_ is the hydrocarbon or phenol quantity in the sample after biological degradation, mg L^−1^. The phenol degradation rate (*Q*_S_, mg L^−1^ h^−1^) over the time period of study was calculated according Pirt (1975).

### Immobilization of bacterial cells on vermiculite

Sorptive immobilization of bacterial cells on vermiculite was performed according to Gong et al. (2016) with some modifications. The expanded vermiculite was washed with distilled water three times and dried at 105 °С. The clay was then ground using a laboratory mill (LZM-1, Olis Ltd., Odessa, Ukraine) and screened through a 200 mesh. After autoclaving, 2.5 g clay powder was added to the flasks containing 100 mL culture broth (to 0.7 OD_540_); flasks were incubated for 3 h at 220 rpm and 28 °C to allow sorption of bacterial cells on the vermiculite. The efficiency of bacterial sorption was assessed by the serial dilution technique with plating on agar medium N53. The vermiculite sediment was washed three times with sterile medium N2 and suspended in sterile physiological saline. Samples were used for enumeration of the bacteria firmly attached to vermiculite after preliminary treatment with a UZD-22 ultrasound disrupter (LLC NPP ‘Akadempribor’, Sumy, Ukraine) set in the following mode: sample processing time twice for 30 s, current strength 0.44 A and frequency 22 kHz.

### Scanning electron microscopy of bacterial cells and their interactions with vermiculite

For scanning electron microscopy, samples of bacterial cells before and after contact with ground vermiculite, where the resulting bacterial-clay sediments were washed twice, were fixed in 2.5%(vol) glutaraldehyde in 0.1 M phosphate buffer, pH 7.2. Samples were then washed twice with phosphate buffer and dehydrated through a 25–100% ascending series of ethanol in distilled water, being left for 20 min at each stage. Two transfers were made in 100% ethanol, and the samples were dried by the critical point method. Air-dried control samples of ground vermiculite were used. The mounted samples were sputter-coated with 30 nm Au/Pd using Gatan Pecs 682 (Gatan Inc., Pleasanton, CA, USA). Scanning electron microscopy (SEM) was performed using a Tescan Mira 3 LMU scanning electron microscope (Tescan, Brno, Czech Republic).

### Statistical analysis

All experiments were performed in triplicate. The obtained data were analysed with Microsoft Office Excel 2010 software standard package.

## Results

### Phenotypic features

**Fig. 1:**
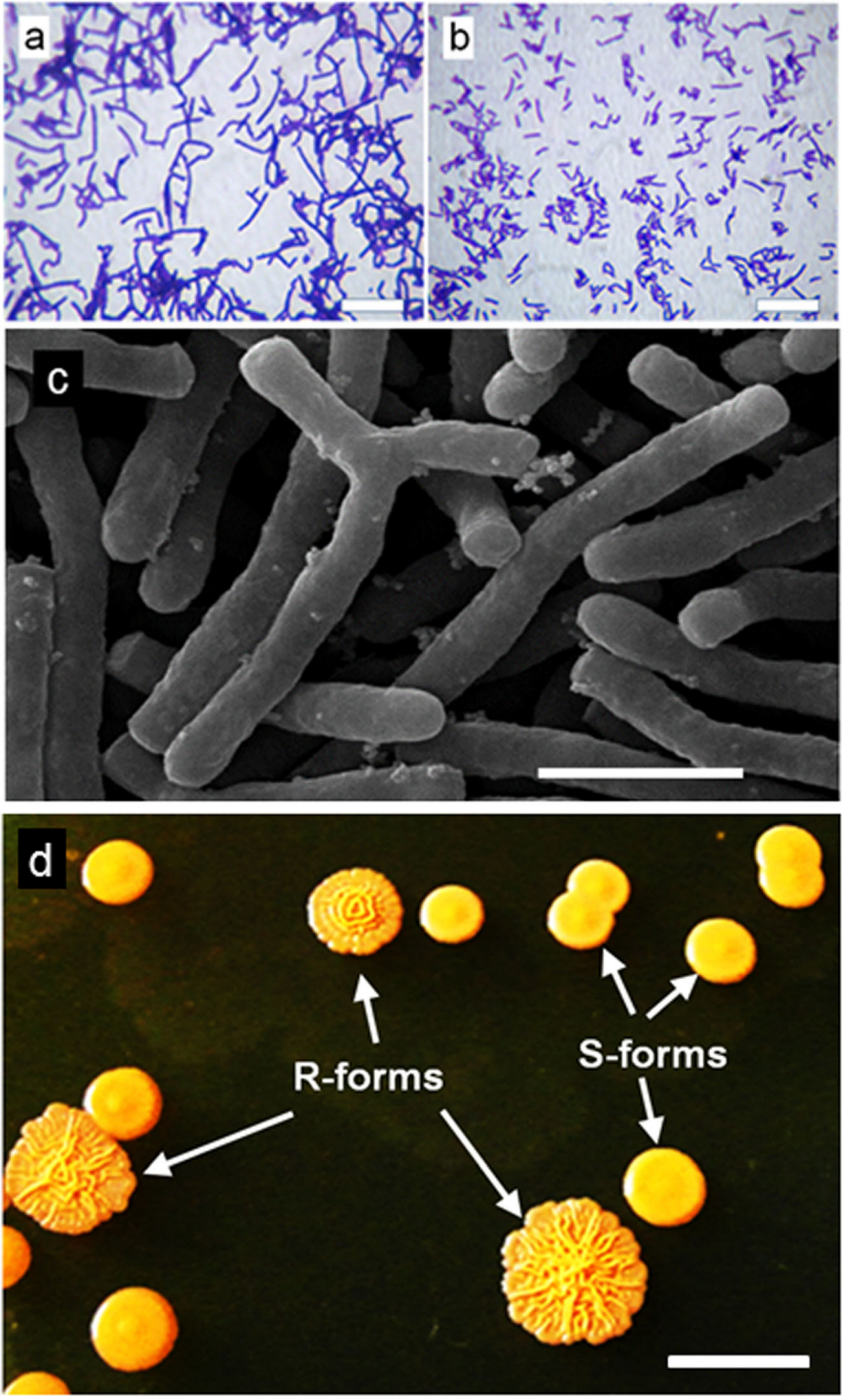
*R*. *aetherivorans* UCM Ac-602 cell morphology. **a**, **b** Light microscopy of Gram-stained cells grown on agar medium N53 for **a** 20 h and **b** 48 h (scale bars = 6 μm). **c** Scanning electron microscopy image of cells grown for 24 h in mineral medium N2 with 750 mg L^−1^ phenol (scale bar = 2 μm). **d** S- and R-form colonies of *R*. *aetherivorans* UCM Ac-602 after 5 days growth on agar medium N53 (scale bar = 3 mm). A typical image is shown from several examinations

Strain UCM Ac-602 was found to be an aerobic, Gram-positive, catalase-positive non-motile actinobacterium which does not form spores and has a rod-coccus life cycle. After 20-h cultivation on solid medium 53, cells were rod-shaped (sometimes irregular) or sometimes showed elementary branching; by 48 h, they fragmented into short rods and coccoid forms (Fig. 1a, b). Cells grown for 24 h in mineral medium N2 containing 750 mg L^−1^ phenol are shown in Fig. 1c. The strain formed pink orange colonies of R- and S-types (Fig. 1d). The organism was able to reduce nitrate and degrade starch but not arbutin, casein or xanthine. The basic physiological and biochemical properties of the strain are shown in Table 1. The strain assimilated phenol and showed a high efficiency in degradation of n-hexadecane (99.6%), kerosene (94.4%) as well as diesel fuel (75.5%). A significantly lower degradation efficiency (56.2%) was observed for assimilation of the transformer oil NITRO 11 GX. During determination of the ring fission mechanism by the Rothera’s reaction, a cell suspension of the strain failed to show the appearance of a yellow colouration in the presence of catechol, indicating absence of the *meta*-aromatic ring cleavage. The appearance of a deep purple colour on testing for *b*-keto-adipic acid confirmed the *ortho*-cleavage of phenol by *R*. *aetherivorans* UCM Ac-602. The chemotaxonomic study of UCM Ac-602 showed that *meso*-diaminopimelic acid was the diagnostic cell wall amino acid while arabinose and galactose were the major cell wall sugars indicating that the cell wall is of chemotype IV (Lechevalier and Lechevalier 1970). The strain contained mycolic acids that co-migrated with those of the type strain of *R*. *rhodochrous* UCM Ac-744 (= DSMZ 43241). The predominant whole-cell fatty acids (> 10%) in UCM Ac-602 grown on liquid medium 53 included C_16:0_ (33.6%), C_18:1_ (29.9%), C_16:1_ (10.8%) and 10-methyl C_18:0_ (9.7%). According to phenotypic properties, strain UCM Ac-602 belongs to actinobacteria of the genus *Rhodococcus* (Jones and Goodfellow 2012).

**Table 1:** Basic physiological and biochemical characteristics of *R*. *aetherivorans* UCM Ac-602

Characteristics	UCM Ас-602
*Cell size*	0.6–1 × 4–6 μm
*Assimilation* at 1% (*w*/*v*) glucose, sucrose, fructose, mannose, melezitose, arabitol, glycerol, sorbitol, mannitol, xylitol	+
*meso*-inositol, α-methyl-d-glucoside, arbutine	−
*Assimilation* at 0.1% (*w*/*v*) sodium lactate, sodium fumarate, sodium benzoate, sodium butyrate, sodium citrate, m-hydroxybenzoic acid	+
sodium gluconate	–
*Degradation tests* starch	+
arbutin, aesculin, xanthine, gelatine, casein, urea hydrolysis, uric acid	–
*Ring fission mechanism*	*ortho*-cleavage of phenol

+ positive reaction; − negative reaction

### Phylogenetic analysis and identification

**Fig. 2:**
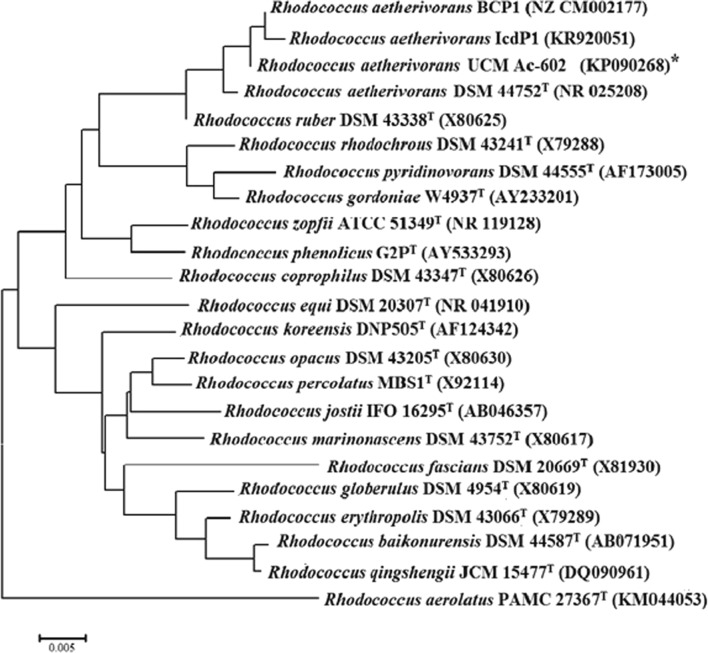
Neighbour-joining phylogenetic tree based on 16S rRNA gene sequences showing the relationship between strain UCM Ac-602 (*) and type strains of representative *Rhodococcus* species. The scale bars represent 5 substitutions per 1000 nucleotide positions. T is the type strain. GenBank accession numbers are given in parentheses

Comparative analysis of the 16S rRNA sequence of strain UCM Ac-602 and those of other closely related taxa retrieved from the GenBank database confirmed the strain belonged to the genus *Rhodococcus* (phylum *Actinobacteria*). The UCM Ac-602 gene sequence was submitted to the GenBank database with the accession number KP090268. The highest level of sequence similarity (100%) was revealed between UCM Ac-602 and type strain *R*. *aetherivorans* DSMZ 44752, followed by *R*. *ruber* DSM 43338 (99.0%), *R*. *rhodochrous* DSMZ 43241 (98.0%) and *R*. *pyridinivorans* DSM 44555 (98.0%). The high level of similarity between these species was noted earlier by Goodfellow et al. (2004), who showed that these species, together with *Rhodococcus coprophilus* and *Rhodococcus zopfii*, formed the *R*. *rhodochrous* subclade. This was confirmed by our phylogenetic analysis (Fig. 2). 16S rRNA sequence similarities between UCM Ac-602 and the type strains of other mycolic-acid-containing taxa varied from 94.0 to 97.0%. The phylogenetic tree determining the position of strain UCM Ac-602 among the species of the *Rhodococcus* genus showed that this strain formed a common cluster with the type strain of *R*. *aetherivorans* 10bc312tip = DSMZ 44752 (NR_025208) and the other strains of this species: *R*. *aetherivorans* BCP1 (NZ CM002177) and IcdP1(KR920051) that demonstrated high catabolic potential in relation to a wide range of alkanes as well as of high molecular weight polycyclic aromatic hydrocarbons and chlorinated compounds (Frascari et al. 2006; Cappelletti et al. 2013; Qu et al. 2015). Phylogenetic analysis placed this cluster within the *R*. *rhodochrous* subclade together with *R*. *ruber* DSM 43338 (X80625) (Fig. 2). The studied strain also differs from *R*. *ruber* and other species belonging to the *R*. *rhodochrous* subclade (Goodfellow et al. 2004) by the complex of diagnostic phenotypic characteristics presented in Table 1. Therefore, based on both genetic and phenotypic studies, it was concluded that strain UCM Ac-602 belongs to the species *R*. *aetherivorans*.

### Phenol biodegradation

**Fig. 3:**
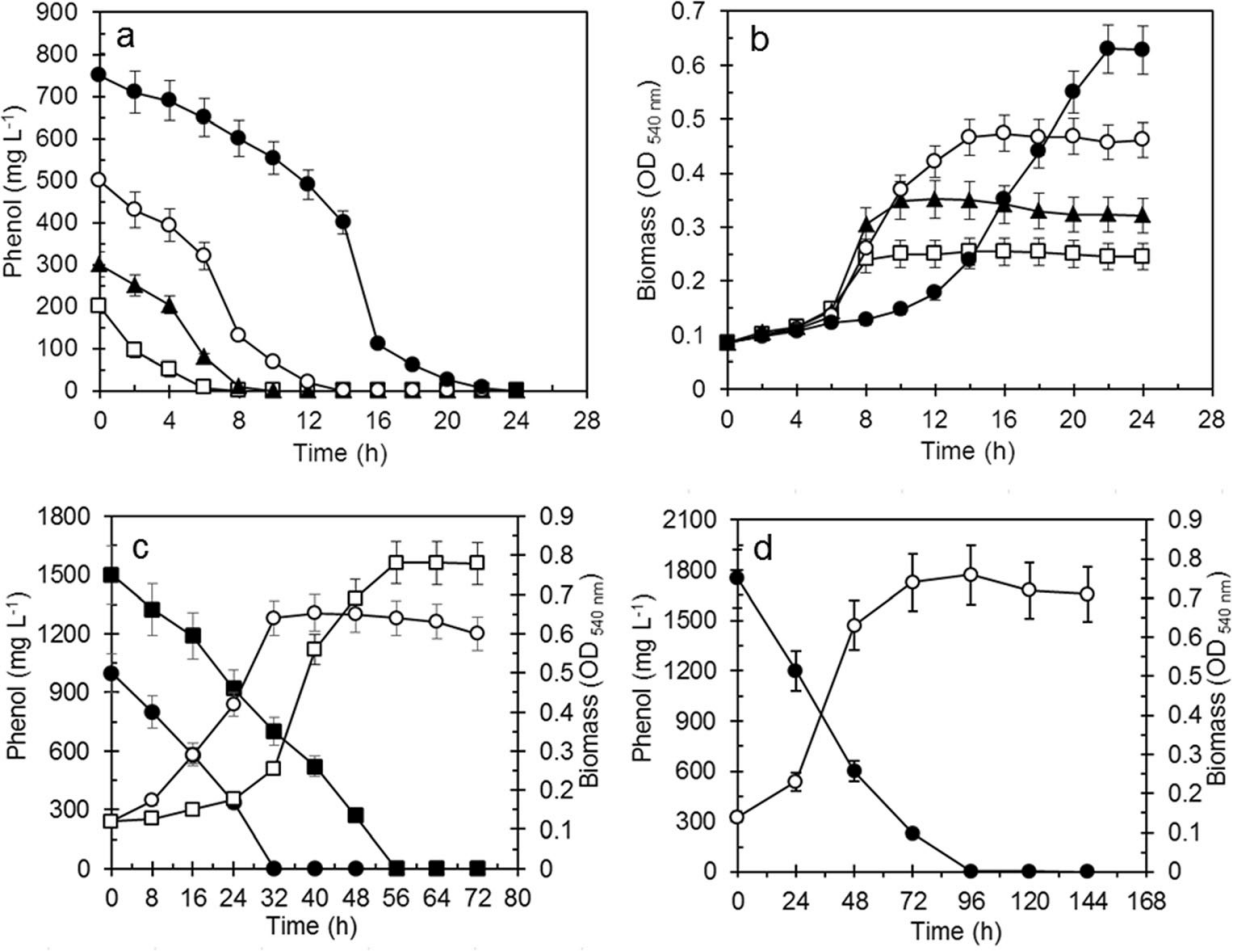
Growth and phenol degradation by freely-suspended cells of *R. aetherivorans* UCM Ac-602. **a** phenol degradation and **b** growth at initial phenol concentrations of (white square) 200 mg L^−1^, (black triangle) 300 mg L^−1^, (white circle) 500 mg L^−1^, (black circle) 750 mg L^−1^. **c** Phenol degradation at an initial phenol concentration of (black circle) 1000 mg L^−1^ and (black square) 1500 mg L^−1^; growth at an initial phenol concentration of (white circle) 1000 mg L^−1^ and (white square) 1500 mg L^−1^. **d** (Black circle) phenol degradation and (white circle) growth at an initial phenol concentration of 1750 mg L^−1^. The error bars indicate standard error of the mean (*n* = 3), and when not shown were less than the symbol dimensions

**Fig. 4:**
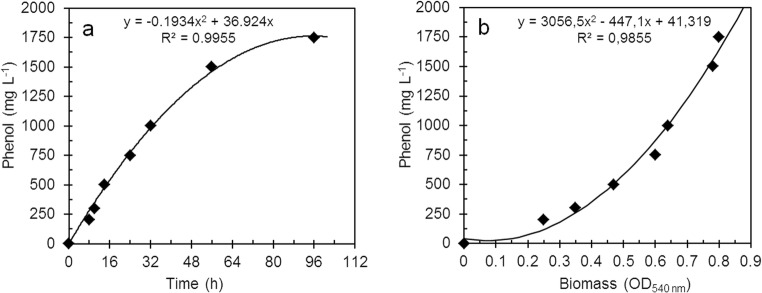
Correlation between **a** phenol concentration and degradation time and **b** phenol concentration and microbial biomass (OD_540_)

Phenol degradation efficiency by suspended cells of *R*. *aetherivorans* UCM Ac-602 was determined by monitoring residual phenol content and growth (Fig. 3). It was observed that the time required for complete degradation and growth at all phenol concentrations increased as a function of the initial substrate concentration. At a concentration of 200 mg L^−1^ (Fig. 3a), residual phenol was absent after 8 h of growth and the OD_540_ value increased more than threefold (Fig. 3b). An increase in phenol concentration to 300, 500 and 750 mg L^−1^ resulted in an increase of the time for complete degradation to 10 h, 14 h and 24 h and OD_540_ values up to 0.33, 0.48 and 0.65, respectively (Fig. 3a, b). At an initial phenol concentration > 750 mg L^−1^, complete assimilation occurred after 32 h at 1000 mg L^−1^ phenol, 56 h at 1500 mg L^−1^ (Fig. 3c) and 96 h at 1750 mg L^−1^ (Fig. 3d). It was established that there was a very strong positive correlation with a second-order polynomial regression for phenol concentration vs. biodegradation time (Fig. 4a) and phenol concentration versus biomass yield (Fig. 4b).

**Fig. 5:**
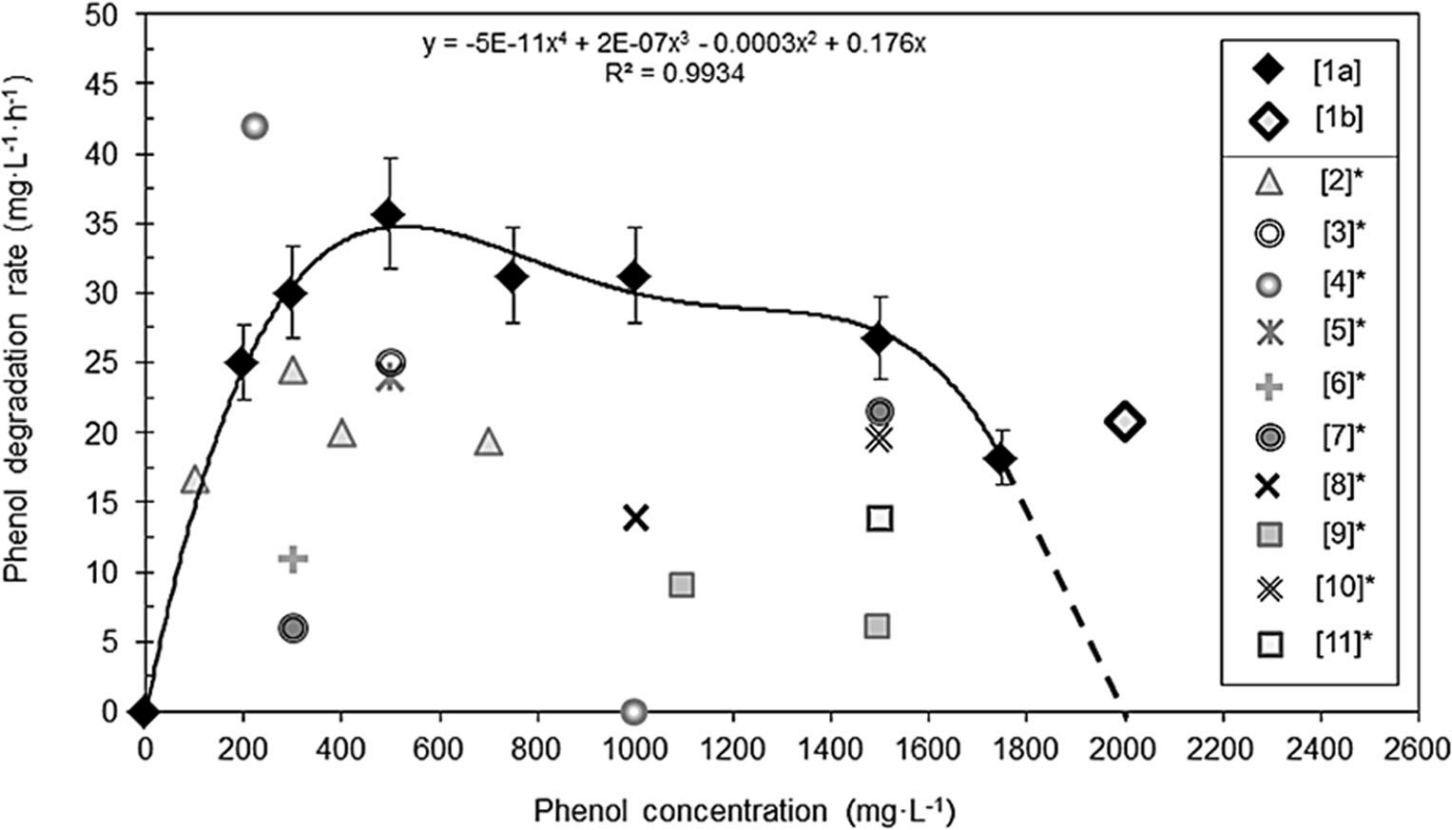
Phenol biodegradation rates exhibited by *R*. *aetherivorans* UCM Ac-602 at different phenol concentrations: [1a] freely-suspended cells and [1b] vermiculite-immobilized cells. The error bars indicate standard error of the mean (*n* = 3), and when not shown were less than the symbol dimensions. The data obtained in this study were compared to biodegradation rate values from the literature as follows: [2] mixed microbial culture (Dey and Mukherjee 2010); [3] *Pseudomonas* sp. SA01 (Mollaei et al. 2010), [4] *R*. *rhodochrous* No21 (Przybulewska et al. 2006), [5] *Rhodococcus* sp. UKMP-5M (Suhaila et al. 2013), [6] *Acinetobacter* sp. (Khleifat 2007), [7] *R*. *opacus* PD630 (Yoneda et al. 2016), [8] *P*. *aeruginosa* SPD 10 (Shweta and Dhandayuthapani 2013), [9] *Rhodococcus* sp. AQ5NOL 2 KCTC 11961BP (Arif et al. 2012), [10] *Acinetobacter* sp. (Adav et al. 2007), [11] *P*. *pseudomallei* NIGB 3 B (Afzal et al. 2007)

The phenol degradation rate (*Q*_S_) increased with the increase of phenol concentration from 200 to 500 mg L^−1^, but then decreased (Fig. 5). The highest *Q*_S_ (35.7 mg L^−1^ h^−1^) was obtained at an initial phenol concentration of 500 mg L^−1^. At an initial phenol concentration of 750 and 1000 mg L^−1^, the *Q*_S_ was 31.2 mg L^−1^ h^−1^. The lowest rate values (less than 30 mg L^−1^ h^−1^) were observed for the lowest and the highest tested initial phenol concentrations (200, 1500 and 1750 mg L^−1^). The dependence of *Q*_S_ on the phenol concentration was described by a fourth-order polynomial regression (*R*^2^ = 0.9934).

**Fig. 6:**
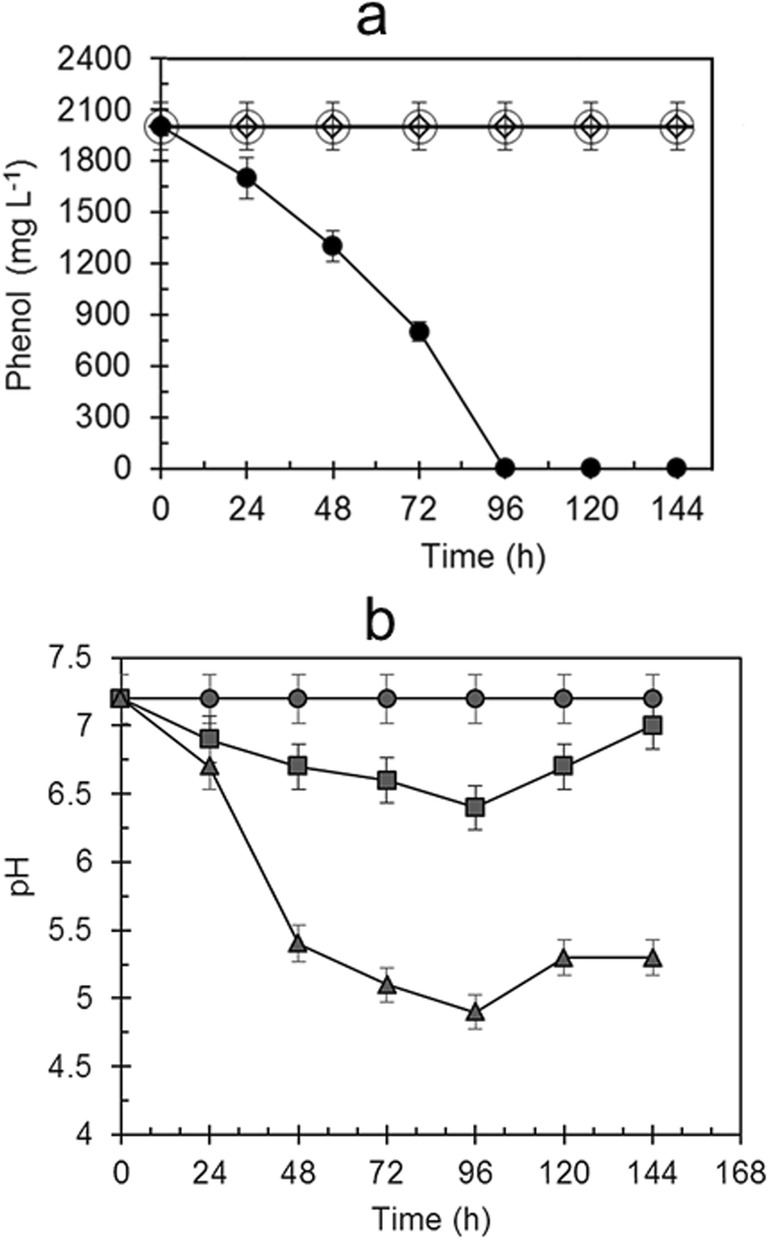
**a** Phenol degradation by (white circle) freely-suspended and (black circle) vermiculite-immobilized *R*. *aetherivorans* UCM Ac-602 cells at an initial phenol concentration of 2000 mg L^−1^, with (white diamond) abiotic control with phenol and vermiculite without the microorganism; and **b** medium pH changes during growth of *R*. *aetherivorans* UCM Ac-602 as (black triangle) freely-suspended cells at a phenol concentration of 1750 mg L^−1^ and (black square) vermiculite-immobilized cells at a phenol concentration of 2000 mg L^−1^; (black circle) pH values for abiotic control medium. The bars indicate standard error of the mean (*n* = 3), and when not shown were less than the symbol dimensions

*R*. *aetherivorans* UCM Ac-602 did not grow when the substrate content in the medium reached 2000 mg L^−1^ (Fig. 6a), but the cells remained viable throughout the entire cultivation period (144 h) which was confirmed by their ability to subsequently grow on agar medium 53. To reduce the inhibitory effect of phenol, cells were immobilized on expanded vermiculite which, perhaps like other clay materials, may then have a positive effect on the ability to utilize high phenol concentrations (Shumkova et al. 2009; Gong et al. 2016). Vermiculite-immobilized cells of *R*. *aetherivorans* UCM Ac-602 were able to grow and completely degrade 2000 mg L^−1^ phenol after 96 h (Fig. 6a) with the *Q*_S_ = 20.8 mg L^−1^ h^−1^ (Fig. 5). In the microorganism-free control, the phenol concentration did not change throughout the experiment proving the absence of interaction between phenol and vermiculite.

In this experiment, the pH value was reduced to 6.5 after 96 h growth when the phenol was completely used (Fig. 6b). Continued cultivation to 144 h was accompanied by an increase in pH to 6.9. In control microorganism-free medium containing only phenol and vermiculite, the pH remained constant at ~ 7.2. In contrast, at the highest initial phenol concentration 1750 mg L^−1^ utilizable by freely-suspended cells, the pH values decreased to 5.0 after 96 h growth when complete degradation of phenol had occurred (Fig. 6b).

**Fig. 7:**
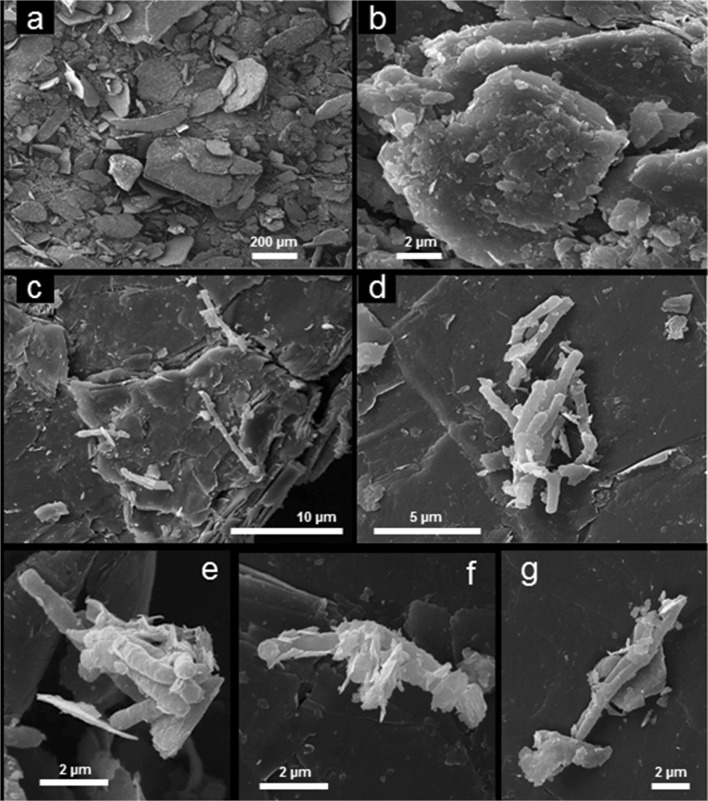
Scanning electron micrographs of *R*. *aetherivorans* UCM Ac-602 cell interactions with vermiculite: **a**, **b** the ground vermiculite used in this study: the size of the crystalline plates varied from 0.2 to 300 μm; **c** bacterial cells attached to the surface of large vermiculite plates; **d**–**g** sorption of small (0.2–2 μm) vermiculite plates on the surfaces of bacterial cells. Typical images are shown from several examinations

The assessment of the interactions between the bacteria and clay mineral showed that the culture broth before cell sorptive immobilization on vermiculite contained 6.1 × 10^9^ CFU mL^−1^ and the amount of free planktonic cells in the supernatant after this process was 2.2 × 10^9^ CFU mL^−1^, indicating that 63.9% of bacterial cells were associated with the vermiculite. The number of cells adherent to the clay assessed by triplicate sediment washing and ultrasonic treatment was 1.8 × 10^8^ CFU gram vermiculite^−1^. SEM examination of the bacterial-clay sediments resulting from the sorptive immobilization process demonstrated that cells were not only adsorbed on the surfaces of large vermiculite particles (Fig. 7c, d) but also seemed to be enveloped by small vermiculite particles (Fig. 7e–g). The aggregation of cells and particles was also often observed (e.g. Fig. 7d, e).

Examination of the effect of fractional phenol addition to the culture medium on the degradation ability of suspended cells of *R*. *aetherivorans* UCM Ac-602 showed that the introduction of 750 mg L^−1^ phenol each 24 h was accompanied by further growth (Table 2). The OD_540_ after 24-h cultivation was 0.65, whereas the fractional introduction of additional phenol every 24 h increased bacterial growth up to OD_540_ = 2.7 (= 2.2 g dry wt L^−1^ biomass) at 96 h. The phenol degradation efficiency at each 24-h interval was in the range 99.7–100% and the cumulative phenol degradation after 96 h reached 2998 mg L^−1^ with specific phenol assimilation being 14.48 nmol g dry wt^−1^).

**Table 2:** The effect of fractional additions of phenol on *R*. *aetherivorans* UCM Ac-602 degradation ability

Time (h)	OD_540_	Phenol assimilation for each 24 h (mg L^−1^)	Degradation efficiency^a^ (%)
0	0.06 ± 0.002	0	0
24	0.65 ± 0.004	750 ± 27.0	100.0
48	1.39 ± 0.006	749 ± 26.1	99.9 ± 1.2
72	1.95 ± 0.009	749 ± 26.0	99.7 ± 2.1
96	2.73 ± 0.014	750 ± 27.1	99.7 ± 2.0

Bacterial cultivation was performed in medium N2; the initial phenol concentration was 750 mg L^−1^; the daily fractional introduction of phenol was 750 mg L^−1^

^a^Degradation efficiency after each 24 h of growth. Data are means ± SD from three independent replicates

## Discussion

Among all the microorganisms listed as efficient phenol assimilators, strains of different *Rhodococcus* species have been found to be highly resistant to a number of toxic xenobiotics, and their ability to degrade many of these compounds has been reported (Martínková et al. 2009). In this study, the actinobacterial strain *R*. *aetherivorans* UCM Ac-602 isolated from lubricant-contaminated soil efficiently degraded phenol as well as n-hexadecane, kerosene, diesel fuel and transformer oil. The variable ability to assimilate phenol has been detected for several different species of genus *Rhodococcus*: *R*. *coprophilus* (Nagamani and Lowry 2009), *R*. *erythropolis* (Soudi and Kolahchi 2011; Korobov et al. 2017; Jha and Veena 2017), *R*. *gordoniae* (Mishra and Lal 2014), *R*. *jostii* (Zidkova et al. 2010), *R*. *opacus* (Shumkova et al. 2009; Yoneda et al. 2016), *R*. *phenolicus* (Rehfuss and Urban 2005), *R*. *pyridinivorans* (Kumari et al. 2013; Al-Defiery and Gopal 2015; Al-Defiery and Reddy 2013; Moghadam et al. 2016), *R*. *rhodnii* (Solyanikova and Golovleva 2015), *R*. *rhodochrous* (Przybulewska et al. 2006), *R*. *ruber* (Pannier et al. 2012) as well as other unidentified strains of *Rhodococcus* sp. (Suhaila et al. 2010, 2013; Arif et al. 2012; Paisio et al. 2012; Hu et al. 2014; Margesin et al. 2005; Nawawi et al. 2017). Evaluation of phenol degradation in such studies was carried out under different varying conditions and with initial phenol concentrations ranging from 100 to 200 up to 2000 mg L^−1^. It is important to note that 1000 mg L^−1^ frequently appears to be the maximum concentration of phenol used in *in vitro* experiments. At higher phenol concentrations, the microorganisms could suffer from substrate inhibition where the growth is inhibited and phenol is not degraded (Prieto et al. 2002). There are very few bacteria that can degrade phenol at an initial concentration above 1500 mg L^−1^ (Nawawi et al. 2014).

Our study, for the first time, has demonstrated the ability of *R*. *aetherivorans* UCM Ac-602 to biodegrade phenol up to 1750 mg L^−1^ through an *ortho*-cleavage pathway, which is a widely distributed mechanism in soil bacteria and fungi (Paisio et al. 2014). This strain is capable of degrading up to 500 mg L^−1^ phenol without any significant inhibition. However, growth inhibition began to increase when the concentration of phenol was ≥ 750 mg L^−1^ with complete growth inhibition of freely-suspended cells at 2000 mg L^−1^.

It should be noted that the often incomplete information on experimental conditions, including media composition, the presence of additional carbon sources, inoculum preparation and adaptation to the toxicant, make a comparative review of available data on phenol degradation by bacteria quite challenging. Therefore, we have analysed only publications that contained clear descriptions of the experimental conditions and quantitative data on phenol degradation (Table 3, Fig. 5).

**Table 3:** *Rhodococcus* spp. and other bacteria able to degrade high phenol concentrations (> 1000 mg L^−1^) in batch culture

Bacterial strain	Isolation source	Free / immobilized cells	Maximum phenol concentration (mg L^−1^)	Cultivation time and other conditions	Temperature (°C)	Phenol degradation (%)	Reference
*R*. *aetherivorans* UCM Ac-602	Lubricant-contaminated soil	Free cells	1500	56 h	28	100	The present work
			1750	96 h			
		Vermiculite immobilized cells	2000	96 h			
*R*. *erythropolis* SKO-1	Oil-polluted soil	Free cells	1200	NА (well-acclimatized cells in the presence of yeast extract)	30	100	Soudi and Kolahchi 2011
*R*. *opacus* 1G	Oil-polluted soil	Vermiculite immobilized cells	1500	48 h	28	100	Shumkova et al. 2009
		Polyacrylamide fibre	1500	24 h		100	
*R*. *opacus* PD630	Soil at a gas works	Free cells	1500	~ 60 h (wild-type strain)	NA^a^	~ 46.7	Yoneda et al. 2016
			1500	~ 60 h (two adapted strains)		~ 86.7	
*R. pyridinivorans* GM3	Soil	Free cells	< 2000	24 h in the presence of yeast extract	32	NA	Al-Defiery and Gopal 2015
*R. pyridinivorans* GM3	Soil	Ca-alginate and polyurethane foam immobilized cells	1500	24 h in mineral medium with yeast extract	32	100	Al-Defiery and Reddy 2013
				24 h in artificial wastewater	32	50.98–61.29	
*R. pyridinivorans* NS1	Activated sludge of a petrochemical effluent	Free cells	1250	72 h in the presence of urea	30	100	Moghadam et al. 2016
			1500–2000	> 72 h in the presence of urea	30	NA	
*Rhodococcus* sp. ad049	Oil-contaminated soil	Free cells	1500	44 h	30	91,9	Hu et al. 2014
*Rhodococcus* sp. UKMP-5 M	Petroleum-contaminated soil	Free cells	1300	10 days	30	0.77	Suhaila et al. 2013
*Rhodococcus* sp. UCC 0009	NA	Free cells	1100	5 days	30	100	Nawawi et al. 2017
			1300	5 days		50	
			1500	12 days		100	
			1800–2100	> 12 days		100	
*Rhodococcus* sp. NO14–1, NO20–3	Petroleum hydrocarbon-contaminated soil	Free cells	1176 (12.5 mM)	25–28 days	10	100	Margesin et al. 2005
			1411 (15 mM)	10–36 days		Residual phenol—11 mM	
*Acinetobacter calcoaceticus*	Phenol-fed aerobic granules	Polyurethane form immobilized cells	1500	76 h	30	100	Adav et al. 2007
*Acinetobacter calcoaceticus* PA	Petrochemical effluent	Free cells	1700	48 h	30	46.2	Liu et al. 2016
*Acinetobacter lowffii* strain UW7	Sludge from coking factory	Free cells	2500	72 h	30	61.1	Liu et al. 2011
*Acinetobacter* sp. AQ5NOL 1	Phenol-contaminated site	Free cells	1100	240 h	30	100	Ahmad et al. 2012
		Encapsulated in gellan gum cells	1100	108 h		100	
			1500	216 h		100	
			1900	240 h		100	
*Bacillus brevis*	Phenol-formaldehyde wastewater	Free cells	1750	~ 132 h	34	100	Arutchelvan et al. 2006
*Bacillus cereus* AKG1 MTCC 9817 AKG2 MTCC 9818	Petroleum refinery and oil exploration site	Free cells	2000	22 days (AKG1)	37	32	Banerjee and Ghoshal 2011
				30 days (AKG2)		20	
		Ca-alginate gel immobilized cells	2000	26 days (AKG1)		54	
				36 days (AKG2)		53	
*Pseudomonas pseudomallei* NIBGE 3B	Pharmaceutical industrial sludge	Free cells	1500	7 days	37	100	Afzal et al. 2007
*Pseudomonas aeruginosa* NIBGE MB			2600	7 days		100	
*Pseudomonas* sp. SA01	Waste water from pharmaceutical plant	Polyvinyl alcohol-alginate	2000	100 h in the presence of thiamine	30	100	Mollaei et al. 2010
		Alginate-chitosan-alginate	2000	110 h in the presence of thiamine		100	

^a^*NA* data not available

When considering the known degradation ability of *Rhodococcus* spp. and other bacteria for phenol concentrations above 1000 mg L^−1^ in terms of (i) the upper limit of the phenol concentrations for assimilation, (ii) the duration of the process, (iii) the extent of phenol degradation and (iv) requirements for additional sources of nutrition and growth stimulants (e.g. yeast extract, urea, thiamine), it is clear that *R*. *aetherivorans* UCM Ac-602 demonstrated one of the best performances for phenol degradation by both suspended and immobilized cells (Table 3).

For freely-suspended cells of *Rhodococcus* sp. UCC 0009, full degradation of phenol at initial concentrations of 1800–2100 mg L^−1^ required more than 12 days (Nawawi et al. 2017), whereas our *R*. *aetherivorans* UCM Ac-602 achieved full phenol assimilation at an initial concentration of 1750 mg L^−1^ after only 4 days. The best phenol degrading performance in terms of highest initial phenol concentrations of 2500 and 2600 mg L^−1^ was recorded for *Acinetobacter lowffii* UW7 with 61.1% degradation after 3 days (Liu et al. 2011) and *Pseudomonas aeruginosa* NIBGE MB with 100% degradation after 7 days (Afzal et al. 2007) (Table 3). The phenol-assimilating activity of freely-suspended cells of our *R*. *aetherivorans* UCM Ac-602 was limited by an initial phenol concentration of 2000 mg L^−1^, although the cells remained viable over 6 days cultivation. For vermiculite-immobilized *R*. *aetherivorans* UCM Ac-602 at an initial phenol concentration of 2000 mg L^−1^, a similar efficiency of phenol degradation (100% degradation over 96 h) was shown to the best reported example, immobilized biomass of *Pseudomonas* sp. SA01 (100% degradation over 100–110 h) (Mollaei et al. 2010). However, in contrast to UCM Ac-602, thiamine addition was required for *Pseudomonas* sp. SA01 phenol degradation (Table 3). The ability of immobilized *Bacillus cereus* strains AKG1 MTCC 9817 and AKG2 MTCC 9818 to degrade phenol at an initial concentration of 2000 mg L^−1^ was significantly lower (53–54% degradation over 26–36 days) compared to our immobilized strain (Banerjee and Ghoshal 2011).

We regard the phenol degradation rate as the best index of the efficiency of phenol degradation and an essential parameter for biotechnological perspectives. Comparative analysis of phenol degradation rates by suspended bacteria at various initial phenol concentrations was carried for our *R*. *aetherivorans* UCM Ac-602 and available data for other bacteria (Fig. 5). It was found that *R*. *aetherivorans* UCM Ac-602 showed excellent phenol degradation ability over a wide range of concentrations with degradation rates significantly exceeding (1.2–5 times) published data for other phenol-assimilating bacteria (Fig. 5). The only exception was a phenol degradation rate of 42 mg L^−1^ h^−1^ reported for *R*. *rhodochrous* No21, at a low initial phenol concentration (225 mg L^−1^), which was ~ 1.5 times greater than the degradation rate for our strain at the same initial concentration. However, *R*. *rhodochrous* No21 lost the ability to grow as the initial phenol concentration increased to 1000 mg L^−1^ (Przybulewska et al. 2006).

Cell immobilization and fractional addition of the toxic substrate are often used to achieve a higher efficiency of phenol degradation and to reduce inhibitory effects (Shumkova et al. 2009; Gong et al. 2016; Al-Defiery and Gopal 2015). For example, cells immobilized on vermiculite or polyacrylamide fibres and phenol fractional addition were used by Shumkova et al. (2009) for *R*. *opacus* 1G strain that could not grow as free cells at phenol concentrations exceeding 750 mg L^−1^ under aerobic batch conditions. The immobilized *R*. *opacus* 1G cells showed complete phenol utilization at 1500 mg L^−1^ over 24–48 h. Fractional phenol introduction at 250 and 500 mg L^−1^ resulted in continuation of culture and an increase in the total utilization of substrate. In contrast to *R*. *opacus* G1, phenol biodegradation by freely-suspended cells of *R*. *aetherivorans* UCM Ac-602 was significantly higher: this strain can grow and assimilate phenol at initial concentrations up to 1750 mg L^−1^. At a fractional daily phenol addition of 750 mg L^−1^, 99.9% of the total added phenol (3000 mg L^−1^) was degraded after 4 days at a high stable rate during all cycles of operation. Furthermore, our data showed that expanded vermiculite was able to improve the phenol degradation efficiency and protect against toxic effects. Numerous studies have shown that interactions of microorganisms with clay adsorbents can lead to an increase in biomass, growth rate and production of enzymes and metabolites (Gadd et al. 2005). Stimulatory effects may arise from the abilities of different clays to serve as (i) pH buffers, (ii) a source of metal cationic nutrients, (iii) specific adsorbents of metabolic inhibitors, other nutrients and growth stimulators and (iv) modifiers of the microbial microenvironment because of their physico-chemical properties such as surface area and adsorptive capacity. As mentioned previously, vermiculite-sorbed *R*. *aetherivorans* UCM Ac-602 grew and completely degraded 2000 mg L^−1^ phenol over 96 h. It should be noted that under the conditions of our experiments, the absence of phenol sorption by natural vermiculite was established. This indicated that a decrease of phenol concentration in the culture broth occurred due to biodegradation by the studied strain, and not due to sorption by vermiculite. Our results are in agreement with other data reporting negligible interactions of natural non-modified clays (vermiculite and bentonite) with phenol (the amount of sorbed phenol was 0.05 mmol g^−1^ of vermiculite) as well as the absence of phenol adsorption on kaolinite (Froehner et al. 2009; Gong et al. 2016). According to Froehner et al. (2009), hydrophobic modification of vermiculite through insertion of hexadecyltrimethylammonium in the mineral interlayer exhibited a high phenol sorption capacity (0.45 mmol g^−1^). One of the possible reasons for vermiculite-sorbed *R*. *aetherivorans* UCM Ac-602 cells to completely degrade 2000 mg L^−1^ phenol may be partially due to the buffering effect of vermiculite leading to less significant changes in medium pH than for freely-suspended cells. The stabilization of pH in a phenol degradation medium in the presence of another clay mineral, kaolinite, was observed by Gong et al. (2016). The buffering effects of clay minerals are likely to be attributable to adsorption of acidic intermediate products of phenol oxidation. Also observed in this study were the dual interactions between *R*. *aetherivorans* UCM Ac-602 cells and vermiculite that included both sorption of small particles of vermiculite to the cells and cell attachment to the large mineral plates which resembled observations of the phenol degrading bacterium *Sphingomonas* sp. GY2B with kaolinite reported by Gong et al. (2016).

In conclusion, the new bacterial isolate from lubricant-contaminated soil, UCM Ac-602, identified as *Rhodococcus aetherivorans*, demonstrated one of the most efficient phenol degradation rates compared to currently known strains of phenol-degrading bacteria. The phenol-degrading ability of *R*. *aetherivorans* UCM Ac-602 has been quantitatively characterized as well as its high tolerance to and ability to assimilate phenol and various hydrocarbons. This organism showed phenol degradation over a very wide range of concentrations, up to 2000 mg L^−1^, and the degradation rate generally considerably exceeded values reported for almost all other bacterial strains. In addition, *R*. *aetherivorans* UCM Ac-602, unlike many other efficient phenol-degrading bacteria, did not require additional sources of nutrition and growth stimulants for phenol assimilation. *R*. *aetherivorans* UCM Ac-602 is clearly a promising candidate for bioremediation approaches for phenol-contaminated environments and process streams. The research also contributes further understanding of the *Rhodococcus* genus as important xenobiotic degrading organisms.
